# Associations between occupational indicators and total, work-based and leisure-time sitting: a cross-sectional study

**DOI:** 10.1186/1471-2458-13-1110

**Published:** 2013-12-01

**Authors:** Corneel Vandelanotte, Mitch J Duncan, Camille Short, Matthew Rockloff, Kevin Ronan, Brenda Happell, Lee Di Milia

**Affiliations:** 1 Institute for Health and Social Science Research, Building 18, Bruce Highway, 4702 Rockhampton, Queensland, Australia

**Keywords:** Sitting time, Sedentary behaviour, Occupation, Leisure-time, Shift-work, Workplace, Full-time, Part-time, Blue-collar, White-collar, Physical demand

## Abstract

**Background:**

A better understanding of how occupational indicators (e.g. job type, doing shift-work, hours worked, physical demand) influence sitting time will aid in the design of more effective health behaviour interventions. The aim of the study was to examine the associations between several occupational indicators and total, occupational and leisure-time sitting.

**Methods:**

Cross-sectional self-report data was collected in November 2011 from 1194 participants through a telephone interview in regional Queensland, Australia (response rate was 51.9%). The Workforce Sitting Questionnaire was used to measure sitting time. Multiple logistic regression was applied to examine associations between sitting time and occupational indicators.

**Results:**

Of all participants 77.9% were employed full-time, 72.7% had white-collar jobs, 35.7% were engaged in shift-work, 39.5% had physically demanding jobs, and 53.2% had high total sitting time (>8 hours a day). Those in physically demanding and blue-collar occupations were less likely to report high total (physically demanding: OR = 0.41,95% CI = 0.29–0.58; blue-collar: OR = 0.55,95% CI = 0.37–0.82) and occupational (physically demanding: OR = 0.26,95% CI = 0.14–0.24; blue-collar: OR = 0.32,95% CI = 0.21–0.49) sitting time compared to those in physically undemanding and white-collar occupations respectively. Working more than 8 hours per day was inversely associated with high leisure-time sitting (OR = 0.44,95% CI = 0.29–0.68). No evidence for ‘compensation’ effects, where lower occupational sitting is compensated with higher leisure-time sitting, was found.

**Conclusions:**

Behaviour change interventions are needed to reduce sitting time as a means to prevent chronic disease. Workplace initiatives to reduce sitting time may be particularly important among individuals employed in white-collar and physical undemanding occupations, although other intervention strategies targeting leisure-time sitting are also required.

## Background

Prolonged sitting time is increasingly recognised as a distinct health risk behaviour associated with increased cardiovascular disease, diabetes, obesity and mortality independent from physical activity levels [1-4]. Therefore, even though adults may meet physical activity recommendations for good health, they may still sit for extended periods each day with detrimental effects on their physical and mental health [5,6]. High levels of sitting time have been reported, with generally over 50% of waking time spent sitting in adults [7,8]. Hence, there is a need to develop effective health behaviour change interventions to reduce sitting time. To succeed in this task the correlates that drive sitting behaviour need to be well understood [9].

A group of seldom studied sitting time correlates include occupational indicators, such as working full or part-time, working normal or shift-work hours, working in a white- or blue-collar job or having a low or highly physically demanding job [10]. It is important to examine whether occupational indicators influence different domains of sitting time and what effect they have on total sitting time, as working adults spend up to half of their workday sitting [11,12], and additionally spend many hours of sitting in their leisure-time (e.g. TV viewing) [13,14]. For example, employees with white-collar jobs and those that are desk-bound are more likely to have high occupational sitting [10,11], and sit up to 75% of their working time [15,16]. However, occupational sitting has been shown to vary according to different occupations, and no studies have simultaneously evaluated the influence of a wide range of different occupational indicators on different domains of sitting (total, occupational and leisure-time). Most studies have focused on a single occupational indicator and a single domain of sitting time; hence more research in this area is needed.

The relationship between occupational sitting and leisure-time sitting is also unclear. Some studies indicate that there is no relationship between the amount of time spent sitting at work and the amount of time spent sitting during leisure-time [11,17], whereas others reported that sedentary workers are more likely to have high leisure-time sitting [18]. Similarly, some studies indicate no relationship between the level of physical demand at work and leisure-time sitting [17], whereas others find that high physically demanding jobs lead to more sitting during leisure-time [19,20]. Accordingly, these latter studies argue that there are ‘compensation effects’ whereby little occupational sitting is compensated with more leisure-time sitting. The inconsistency of these findings suggest the need for further research.

Given that most adults in Western nations spend the most of their lives employed [21], it is important to clarify the influence and interactions that occupational indicators have on different domains of sitting time [10]. This information can then be utilised to direct the development of public health interventions [22]. Therefore, the aim of this study was to examine the associations between several occupational indicators and total, occupational and leisure-time sitting.

## Methods

### Sample

Cross-sectional self-report data were obtained from the Healthy Shift Worker Study conducted for the Institute for Health and Social Science Research by the Population Research Laboratory (PRL) at CQUniversity, in November of 2011. The Computer Assisted Telephone Interview (CATI) surveys were conducted by trained interviewers. A two-staged sampling design was used to select households (random-digit-dialling of landline telephones) and individuals (one employed person, 18 years of age or older, within each household) in three distinct geographical areas in Queensland Australia (the Gladstone, Mackay and Rockhampton Regional Council areas were selected because of the high proportion of shift workers living in these areas). If the interviewers were unsuccessful in establishing contact on their first call, a minimum of five call-back attempts were made. Participation was voluntary, confidential and remuneration was not paid. Informed consent was obtained from participants prior to conducting the survey and approval by the Human Research Ethics Committee of Central Queensland University Australia was obtained.

### Measures

The telephone survey was pilot-tested by trained interviewers with 71 randomly-selected households. Interviewer comments (e.g. confusing wording, inadequate response categories, question order effect, etc.), respondent feedback, average interview times and pre-test frequency distributions were reviewed before modifications were made to the questionnaire. The survey was approximately 40 minutes in duration and assessed socio-demographics, dietary habits, physical activity, sitting time, psychological well-being and health. Only the details of the measures used in this study are presented below.

Sitting time was measured using the Workforce Sitting Questionnaire, which has demonstrated acceptable levels of validity in a sample of Sydney employees that were predominantly female (63.2%), university educated (64.2%), full-time employed (73.4%), between 18–49 years of age (81%) and of normal weight (61.1%) [23]. This 10-item questionnaire measures sitting time during work, commuting to/from work, using a computer at home, watching TV and other leisure-time activities for work and non-work days during the last 7 days. Total sitting time was defined as the sum of sitting time in all domains for all days. Participants were categorised as achieving high total sitting when they were sitting for more than 8 hours on average every day, as this threshold has been associated with adverse health outcomes [1,2]. To calculate total leisure-time sitting the categories ‘using a computer at home’, ‘watching TV’ and ‘other leisure-time activities’ were summed. A median split was used to categorise participants as achieving high or low levels of occupational (+/− 120 min/day) and leisure-time (+/− 240 min/day) sitting time.

Physical activity over the last 7 days was measured using the long form, telephone administered, International Physical Activity Questionnaire (IPAQ long). This 31-item questionnaire measures physical activity at work, transportation, household, and leisure-time. For each topic in each category, respondents reported the number of days and the number of minutes per day spent undertaking the activity. A total physical activity score, expressed in minutes of activity per week, was obtained when all questions were summed. The IPAQ has been shown to be a valid and reliable instrument to measure physical activity among adults at a population level [24]. Standard IPAQ protocols were used to analyse the data and categorise participants as achieving high or low levels of total physical activity [25].

Occupational indicators included working full or part time; having a white- (includes professional occupations) or blue-collar job [26]; having worked in the same organisation more or less than 5 years; being a shift-worker (working between 6 pm and 7 am) or not; working night shifts or not; working rotational shifts or not; working more or less than 40 hours on a usual week; working more or less than 8 hours on a usual day; and having a job with high or low physical demand [27]. Demographic variables included gender, age (above or below the age of 45), Body Mass Index (BMI = (weight (kg)/(height (cm))^2^), educational attainment (more or less than high school education) and family income (over or under $100,000AUD per year).

### Analyses

Descriptive statistics (Chi^2^ and *t*-tests) and multiple logistic regression were applied to identify differences and associations between a range of occupational indicators and sitting time. Bivariate and multiple logistic regression models were used to determine the association between occupational indicators and sitting variables. Each multiple logistic regression model included all occupational indicators as well as the demographic variables described above to adjust for confounding effects as these variables have previously shown to be of influence on sitting levels [28]. All analyses were conducted in 2013, Alpha levels were set at 0.05, IBM SPSS Statistics version 20 was used to conduct the statistical tests.

## Results

In total 2323 households were contacted for this study and 1194 participated (a response rate of 51.9%). The proportion of male (46.1%) and female (53.9%) respondents was reasonably similar (see Table 1). The average BMI was 28.0 (±5.6) and 66% were classed as overweight or obese, which is comparable to national Australian data [29]. The average participant age was 45.3 (±11.2) years, and the majority of participants (73.8%) had at least a high school education. The majority of participants were employed full time (77.9%) in white-collar jobs (72.7%). About one third of the sample was engaged in shift work (35.1%), in a high physically demanding job (39.5%) and/or were working more than 40 hours per week (35.7%). Over half of the sample had a high total sitting time (53.2%) and worked in the same organisation for more than 5 years (55.7%).

**Table 1:** Participant characteristics

	**Total group**	**Males**	**Females**
	**N (%)**	**N (%)**	**N (%)**
**Personal characteristics**			
Sex			
Male	551 (46.1)	-	-
Female	643 (53.9)	-	-
Age			
< 45 years	561 (47.0)	260 (47.2)	301 (46.8)
≥ 45 years	633 (53.0)	291 (52.8)	342 (53.2)
Education			
Up to high school	292 (26.2)	130 (25.0)	162 (27.2)
More than high school	823 (73.8)	390 (75.0)	433 (72.8)
Family Income			
< $100,000 AUD	333 (38.3)	147 (34.8)	186 (41.7)
≥ $100,000 AUD	536 (61.7)	276 (65.2)	260 (58.3)
Body Mass index (BMI)			
≤ 25	351 (30.7)	118 (21.4)	233 (36.2)
> 25	792 (66.3)	422 (76.6)	370 (57.5)
**Job characteristics**			
Full time job	926 (77.9)	506 (92.0)	420 (65.7)
Part-time job	263 (22.1)	44 (8.0)	219 (34.3)
White-Collar job	834 (72.7)	288 (54.0)	546 (88.9)
Blue-Collar job	313 (27.3)	245 (46.0)	68 (11.1)
< 5 years in same org	217 (44.3)	225 (41.8)	292 (46.4)
≥ 5 years in same org	650 (55.7)	313 (58.2)	337 (53.6)
Non shift worker	775 (64.9)	293 (53.2)	482 (75.0)
Shift-worker	419 (35.1)	258 (46.8)	161 (25.0)
No night shifts	925 (77.5)	369 (67.0)	556 (86.5)
Does night shifts	269 (22.5)	182 (33.0)	87 (13.5)
No rotational shifts	983 (82.3)	399 (72.4)	584 (90.8)
Does rotational shifts	211 (17.7)	152 (27.6)	59 (9.2)
≤ 40 hrs/wk of work	768 (64.3)	227 (41.2)	541 (84.1)
> 40 hrs/wk of work	426 (35.7)	324 (58.8)	102 (15.9)
≤ 8 hrs/day of work	643 (53.9)	165 (29.9)	478 (74.3)
> 8 hrs/day of work	551 (46.1)	386 (70.1)	165 (25.7)
Low physical demand	721 (60.5)	318 (57.8)	403 (62.8)
High physical demand	471 (39.5)	232 (42.2)	239 (37.2)
**Total sitting time**			
< 8 hours/day	549 (46.8)	230 (42.6)	319 (50.5)
≥ 8 hours/day	623 (53.2)	310 (57.4)	313 (49.5)

Table 2 provides an overview of total, occupational and leisure-time sitting according to occupational indicators. As illustrated, leisure-time sitting contributes substantially more to the calculation of total sitting time compared to occupational sitting time across all occupational indicators. Differences between occupational categories and the respondents’ leisure-time sitting were relatively small, whereas they were often large for occupational sitting time; especially for the high/low physical demand and blue/white-collar job categories. There were few differences between occupational categories for total sitting time, although when present they were driven by the differences reported in occupational sitting time. Differences between occupational indicators for leisure-time sitting had no or little influence on total sitting time.

**Table 2:** Total, occupational and leisure-time sitting according to main occupational indicators (Mean (±SD))

	**Total sitting time (min/day)**^**a**^		**Occupational sitting time (min/day)**		**Leisure-time sitting (min/day)**	
	**Mean (±SD)**	***t*-test**	**Mean (±SD)**	***t*-test**	**Mean (±SD)**	***t*-test**
**Total Group**	530.9 (±251.3)	--	168.9 (±172.2)	--	318.4 (±257.7)	--
**General**						
Full-time job	543.5 (±254.2)	3.1***	179.9 (±180.1)	4.2***	322.2 (±258.8)	0.9
Part-time job	487.3 (±236.7)		129.3 (±133.9)		304.6 (±253.1)	
White-collar job	546.6 (±243.7)	3.8***	200.4 (±172.3)	10.3***	314.2 (±256.3)	0.3
Blue-collar job	482.7 (±262.9)		87.7 (±145.0)		319.7 (±260.7)	
< 5 years in same org	519.5 (±248.5)	1.6	149.4 (±166.9)	3.5***	326.6 (±261.9)	0.6
≥ 5 years in same org	544.6 (±254.0)		184.4 (±175.9)		317.3 (±257.4)	
**Shift**						
Non shift worker	536.8 (±243.4)	1.1	185.1 (±166.1)	4.4***	319.1 (±259.2)	0.1
Shift-worker	519.8 (±265.1)		138.7 (±179.7)		317.0 (±255.4)	
No night shifts	529.8 (±248.1)	0.2	180.2 (±169.7)	4.2***	313.1 (±254.9)	1.3
Does night shifts	534.7 (±262.3)		129.7 (±175.2)		336.5 (±266.9)	
No rotational shifts	529.2 (±247.9)	0.4	179.5 (±172.0)	4.6***	313.8 (±255.5)	1.3
Does rotational shifts	538.6 (±267.0)		119.5 (±172.0)		339.7 (±267.3)	
**Demand**						
≤ 40 hrs/wk of work	537.8 (±249.9)	0.7	161.3 (±162.3)	2.0*	334.9 (±270.0)	2.9**
> 40 hrs/wk of work	527.0 (±252.1)		182.6 (±187.7)		288.6 (±231.4)	
≤ 8 hrs/day of work	529.7 (±248.9)	0.1	159.4 (±158.8)	2.1*	335.1 (±265.9)	2.4*
> 8 hrs/day of work	532.7 (±254.3)		180.0 (±186.2)		298.8 (±246.7)	
Low physical demand	580.5 (±234.7)	8.5***	220.2(±176.6)	13.5***	325.4 (±260.9)	1.1
High physical demand	456.4 (±257.5)		91.0 (±131.7)		307.8 (±253.3)	

^a^Total sitting time is the sum of sitting during occupation, leisure-time and transport, the latter category was not reported;

*P < 0.05; **P < 0.01, ***P < 0.001; min/day = minutes per day, hrs/wk = hours per week, hrs/day = hours per day.

For the unadjusted models, Table 3 shows that the majority of associations were significant for occupational sitting time, fewer were significant for total sitting time and almost none were significant for leisure-time sitting. However, as also reported in Table 3, few outcomes were significant in the fully adjusted models for all domains of sitting time. Participants with high physically demanding jobs were less likely to report high total sitting time when compared to those with a low physically demanding job (OR = 0.41, 95% CI 0.29–0.58); and they were also less likely to report high occupational sitting time (OR = 0.26, 95% CI 0.14–0.24). Similarly, blue-collar workers were less likely to report high total (OR = 0.55, 95% CI 0.37–0.82) and occupational (OR = 0.32, 95% CI 0.21–0.49) sitting time when compared to white-collar workers. Further, participants employed part-time (OR = 0.64 95% CI 0.42–0.98) were less likely to report high total sitting time, compared to those employed full-time. The only significant outcome on leisure-time sitting was for those who work more than eight hours per day on working days; they were less likely to have high leisure-time sitting (OR = 0.44 95% CI 0.29–0.68) compared to those working less than eight hours a day.

**Table 3:** Odds of having high levels of total, occupational and leisure-time sitting according to occupational indicators

	**High total sitting time**	**High total sitting time**	**High occupational sitting time**	**High occupational sitting time**	**High leisure-time sitting**	**High leisure-time sitting**
	**(Bivariate)**	**(Multiple)**^**a**^	**(Bivariate)**	**(Multiple)**^**b**^	**(Bivariate)**	**(Multiple)**^**c**^
**General**						
Full-time job	1	1	1	1	1	1
Part-time job	**0.56 (0.43 – 0.75)**	**0.64 (0.42 – 0.98)**	0.82 (0.62 – 1.08)	0.67 (0.42 – 1.05)	0.96 (0.73 – 1.27)	0.97 (0.64 – 1.48)
White-collar job	1	1	1	1	1	1
Blue-collar job	**0.61 (0.47 – 0.80)**	**0.55 (0.37 – 0.82)**	**0.20 (0.15 – 0.27)**	**0.32 (0.21 – 0.49)**	1.08 (0.83 – 1.40)	1.00 (0.68 – 1.47)
< 5 years in same org	1	1	1	1	1	1
≥ 5 years in same org	1.15 (0.91 – 1.45)	0.95 (0.68 – 1.32)	**1.62 (1.28 – 2.04)**	1.28 (0.89 – 1.84)	0.90 (0.72 – 1.14)	0.78 (0.56 – 1.08)
**Shift**						
Non shift-worker	1	1	1	1	1	1
Shift-worker	1.01 (0.79 – 1.28)	1.14 (0.71– 2.29)	**0.42 (0.33 – 0.54)**	0.80 (0.48 – 1.34)	1.00 (0.79 – 1.27)	0.78 (1.49 – 1.24)
No night shifts	1	1	1	1	1	1
Does night shifts	1.11 (0.84 – 1.46)	1.38 (0.78 – 2.45)	**0.42 (0.31 – 0.56)**	0.76 (0.41 – 1.44)	1.24 (0.94 – 1.63)	1.32 (0.75 – 2.32)
No rotational shifts	1	1	1	1	1	1
Does rotational shifts	1.11 (0.82 – 1.50)	1.12 (0.62 – 2.02)	**0.40 (0.30 – 0.55)**	0.72 (0.38 – 1.38)	1.21 (0.89 – 1.63)	1.56 (0.88 – 2.78)
**Demand**						
≤ 40 hrs/wk of work	1	1	1	1	1	1
> 40 hrs/wk of work	1.19 (0.93 – 1.51)	0.82 (0.54 – 1.27)	0.97 (0.77 – 1.23)	0.76 (0.47 – 1.21)	**0.73 (0.57 – 0.93)**	0.82 (0.54 – 1.24)
≤ 8 hrs/day of work	1	1	1	1	1	1
> 8 hrs/day of work	1.08 (0.86 – 1.36)	0.82 (0.54 – 1.26)	0.94 (0.75 – 1.18)	1.57 (0.99 – 2.49)	**0.62 (0.49 – 0.78)**	**0.44 (0.29 – 0.68)**
Low physical demand	1	1	1	1	1	1
High physical demand	**0.37(0.29 – 0.47)**	**0.41 (0.29 – 0.58)**	**0.18 (0.14 – 0.24)**	**0.26 (0.18 – 0.37)**	0.85 (0.67 – 1.07)	1.02 (0.71– 1.45)

^a^multiple logistic regression model adjusted for gender, age, education, income, BMI, physical activity and all work variables.

^b^multiple logistic regression model adjusted for gender, age, education, income, BMI, physical activity, leisure-time sitting and all work variables.

^c^multiple logistic regression model adjusted for gender, age, education, income, BMI, physical activity, occupational sitting time and all work variables.

Values in **bold** indicate a significant odds ratio.

Table 4 reports on the odds of having high levels of total, occupational and leisure-time sitting when examining two occupational indicators in combination. This Table highlights the relative importance of having a physically demanding job, followed closely by having a blue-collar job, over the other occupational indicators in terms of having lower sitting time. Participants with a physically demanding job were less likely to have high total and occupational sitting time irrespective of whether they had a part- or full-time job, a white- or blue-collar job, or a shift-work or non shift-work job. To a smaller extent this was similar for people with a blue-collar job. However, no significant odds ratios were found for leisure-time sitting in relation to all examined occupation indicators. Further, participants who did not have a shift-work job were less likely to have high total and occupational sitting when they were engaged in a part-time, blue-collar or physically demanding job compared to those who were engaged in full-time, white-collar or physically not demanding job.

**Table 4:** **Odds of having high levels of total, occupational and leisure-time sitting when examining two occupational indicators in combination**^**a**^

	**High total sitting time**		**High occupational sitting time**		**High leisure-time sitting**	
	**Full-time job**	**Part-time job**	**Full-time job**	**Part-time job**	**Full-time job**	**Part-time job**
White-collar job	1	1	**1**	1	1	1
Blue-collar job	**0.49 (0.32 – 0.75)**	1.18 (0.43 – 3.22)	**0.33 (0.21 - 0.50)**	**0.16 (0.04 – 0.58)**	0.85 (0.57 – 1.27)	1.48 (0.56 – 3.91)
Non shift-worker	1	1	1	1	1	1
Shift-worker	1.28 (0.87 – 1.87)	1.95 (0.85 – 4.51)	**0.60 (0.40 – 0.90)**	0.69 (0.28 – 1.71)	1.04 (0.72 – 1.49)	0.58 (0.26 – 1.32)
Low physical demand	**1**	1	**1**	**1**	1	1
High physical demand	**0.39 (0.26 – 0.57)**	0.56 (0.26 – 1.21)	**0.26 (0.17 – 0.38)**	**0.29 (0.13 – 0.64)**	0.77 (0.54 – 1.13)	1.17 (0.57 – 2.42)
	**Blue-collar job**	**White-collar job**	**Blue-collar job**	**White-collar job**	**Blue-collar job**	**White-collar job**
Full-time job	1	1	1	1	1	1
Part-time job	1.51 (0.57 – 4.03)	**0.57 (0.37 – 0.89)**	0.43 (0.12- 1.46)	0.80 (0.50 – 1.29)	1.27 (0.51 – 3.21)	0.88 (0.57 – 1.36)
Non shift-worker	1	1	1	1	1	1
Shift-worker	1.76 (0.97 – 3.19)	1.21 (0.79 – 1.85)	1.01 (0.53 – 1.95)	**0.51 (0.33 – 0.80)**	0.77 (0.44 – 1.36)	0.99 (0.66 – 1.47)
Low physical demand	**1**	**1**	**1**	**1**	1	1
High physical demand	**0.34 (0.19 – 0.64)**	**0.44 (0.29 – 0.66)**	**0.34 (0.17 – 0.66)**	**0.23 (0.15 – 0.36)**	1.11 (0.62 – 1.97)	0.74 (0.50 – 1.11)
	**Shift-worker**	**Non shift-worker**	**Shift-worker**	**Non shift-worker**	**Shift-worker**	**Non shift-worker**
Full-time job	1	1	1	1	1	1
Part-time job	1.28 (0.55 – 2.98)	**0.55 (0.34 – 0.88)**	1.19 (0.19 – 2.87)	**0.60 (0.35 – 0.99)**	0.70 (0.31 – 1.55)	1.04 (0.66 – 1.63)
White-collar job	1	1	1	**1**	1	1
Blue-collar job	0.57 (0.32 – 1.02)	**0.49 (0.29 – 0.82)**	**0.32 (0.17 – 0.59)**	**0.24 (0.13 – 0.42)**	0.78 (0.46 – 1.32)	1.02 (0.81 – 1.69)
Low physical demand	**1**	**1**	**1**	**1**	1	1
High physical demand	**0.36 (0.21 – 0.61)**	**0.48 (0.31 – 0.74)**	**0.29 (0.17 – 0.52)**	**0.26 (0.16 – 0.41)**	0.82 (0.49 – 1.37)	0.85 (0.55 – 1.31)
	**High physical demand**	**Low physical demand**	**High physical demand**	**Low physical demand**	**High physical demand**	**Low physical demand**
Full-time job	1	1	1	**1**	1	1
Part-time job	0.83 (0.43 – 1.60)	0.62 (0.37 – 1.03)	0.90 (0.44 – 1.83)	**0.67 (0.38 – 1.16)**	1.13 (0.61 – 2.13)	0.85 (0.51 – 1.40)
White-collar job	1	1	**1**	**1**	1	1
Blue-collar job	0.60 (0.34 – 1.08)	**0.52 (0.31 – 0.87)**	**0.35 (0.18 – 0.69)**	**0.27 (0.16 – 0.44)**	1.25 (0.71 – 2.19)	0.79 (0.48 – 1.28)
Non shift-worker	1	1	1	1	1	1
Shift-worker	0.39 (0.74 – 2.13)	1.45 (0.92 – 2.30)	0.63 (0.35 – 1.16)	0.65 (0.41 – 1.03)	0.88 (0.53 – 1.46)	0.97 (0.64 – 1.49)

^a^This Table reports on outcomes of 24 different multiple logistic regression models adjusted for relevant variables such as gender, age, education, income, BMI, occupational sitting time, leisure-time sitting time, physical activity and all work variables.

Values in **bold** indicate a significant odds ratio.

## Discussion

The occupational indicators with the strongest influence on both total and occupational sitting time were having a physically demanding job and having a blue-collar job, although neither had a significant impact on leisure-time sitting. With the exception of having a part-time job (lower total sitting) and working more than eight hours a day (lower leisure-time sitting), no significant differences were observed for any of the other occupational indicators. Overall, the differences in sitting time between the occupational indicators were smaller than one might expect based on stereotypical notions of differences between occupations [30]. In fact, total sitting time was high across all occupational indicators, as was leisure-time sitting. Compared to this study, several studies report lower minutes of total sitting time a day [11,31,32], although Chau et al. [18], who used the same sitting time measure, reported higher total sitting a day. It is difficult to explain these differences, although the use of different measures and study populations (the sample of Chau et al. [18] was characterised by lower proportions of women, educated participants, full-time and white-collar workers compared to the sample in this study) is likely to have contributed. The total time spent sitting was predominantly made up of time spent in leisure-time sitting (approximately 60%), irrespective of occupational indicators. Few studies have reported on both occupational and leisure-time sitting: a study by Jans et al. reported similar outcomes to our study [11], whereas Chau et al., who’s study predominantly included office workers, reported more occupational than leisure-time sitting [18].

The outcomes for total and occupational sitting time in relation to physically demanding and blue-collar jobs are as one would expect and in line with previous research. In a study by Mummery et al. white-collar employees sat significantly more than blue-collar employees [12]. Similar outcomes regarding blue/withe collar workers were reported in other studies [11,32], and a study by Van Dyck et al. was able to demonstrate the same observation using objective measures (accelerometry) [31]. In relation to physical demand Tigbe et al. demonstrated, in line with the present study, that those in a physically demanding job had lower total and occupational sitting time [17]. There is, however, limited evidence in relation to sitting time and physical demand and therefore a review by Rhodes et al. indicated that sustained research is needed before conclusions can be drawn in this area [28]. The same applies in relation to shift-work, where no studies were found to compare the unexpected outcomes of this study: there were no significant differences in total and occupational sitting time between shift-workers and non shift-workers. A closer look at job-types undertaken by shift- and non shift-workers (not reported) in this study might explain this outcome, as both groups engaged to a large extent in occupations that could be typified by low (e.g. shift-worker: health services; non shift-worker: construction) and high (e.g. shift-worker: transport; non shift-worker: education) sitting time.

The average differences in leisure-time sitting were minimal across occupational indicators; as such, on a group level, this study did not find evidence for ‘compensation effects’: those with blue-collar, shift-work or physically demanding jobs did not sit significantly more in their leisure-time and vice-versa. This is in line with some studies [11,17], but in contrast with studies that indicate that people with blue-collar [33], shift-work [19], and physically demanding jobs [20] sit more in their leisure-time. However, one significant difference was observed for leisure-time sitting: those working more than eight hours per day sat less in their leisure-time. This is not unexpected, as it is likely due to having less leisure-time when performing longer working hours which results in having less opportunity to sit, and this finding is in line with several other studies that report lower leisure-time sitting when working more hours [14,32]. This study illustrates that there is a need to reduce leisure-time sitting irrespective of occupational background, given the high sitting time reported across categories. In this context, the promotion of a positive balance between time spend in light intensity physical activity and sitting time (i.e. spending more time in light intensity physical activity than time spent sitting) is recommended [8,34], as several studies have demonstrated poorer health when the balance between sitting time and light intensity physical activity is negative [35].

Consistent with the current study, previous studies have indicated that those working full-time have higher total sitting when compared to those working part-time [32]. Interestingly, other studies have reported that those who are unemployed sit more than those who have a job [27,31,36]. As such, there seems to be a U-shaped relationship with high sitting in those who work either very little or very much and lower sitting in those who work moderate amounts of time. However, further investigation is needed to determine whether this relationship only applies for those with white-collar employment, but perhaps not for those with blue-collar and/or physically demanding jobs, as the current study seems to suggest. In any case the current study illustrates, in line with previous research [10,11,15], that workplace initiatives to reduce occupational sitting are especially needed for those in white-collar or sedentary occupations, but to a lesser extent for those with blue-collar or physically demanding jobs. Reducing sitting time at work will require innovative approaches that don’t adversely impact on worker productivity.

The main limitation in this study is the use of a cross-sectional design that does not allow inferences about causality; longitudinal data is needed to further study this area of interest. Secondly, all data was obtained through self-report questionnaires. However, the instrument used to measure sitting time has shown good reliability and validity [23], and the total sitting time reported in other studies using this measure was similar to what is reported in this study. Furthermore, the data was obtained through well-trained CATI interviewers, which has shown to produce reliable data [37]. The response rate of 51.9% is acceptable for this type of survey although the potential for non-response bias is acknowledged. Response rates in CATI surveys are declining due to aggressive tele-marketing and increased efforts of people to protect their privacy [38,39]. A strength of the study was the sampling design applied to randomly select a population to participate in this study, however as this study was part of the Healthy Shift Workers Study data were only collected in cities known to have high proportions of shift workers, as such the sample obtained in this study may not be representative for the wider Australian population. Nevertheless, the greater variability in working conditions obtained in the study sample should be considered as a strength. To our knowledge this study is also the first that evaluated a broad range of occupational indicators simultaneously in terms of how they influence different domains of sitting time. Most studies focus on a single occupational indicator and a single domain of sitting, making it more difficult to get an overview and see patterns.

## Conclusion

In conclusion, this study adds some important findings. Firstly, total and leisure-time sitting were high irrespective of occupational indicators, and behaviour change interventions are needed to reduce sitting time and prevent chronic disease. Secondly, white-collar and low physically demanding jobs were significantly associated with high occupational sitting time, and need targeting by workplace initiatives to reduce sitting time. Thirdly, no evidence for ‘compensation’ effects was found; where lower sitting at work is compensated with higher sitting in leisure. Fourth, working full-time is associated with higher total sitting; although working long hours is associated with lower leisure-time sitting. While causal relations can not be inferred from this cross-sectional study, the outcomes suggest it may be beneficial to limit total working hours and avoid working over-time as a way to reduce total sitting. Further, it is recommended to couple this with efforts to increase light intensity physical activity during leisure-time to generate a positive balance with sitting time. Future research should consider the use of experimental and time-series designs to assess whether changes in the different domains of sitting time can be brought about using workplace interventions.

## Competing interests

The authors declare that they have no competing interests.

## Authors’ contributions

CV wrote the manuscript and conducted the statistical analyses, MJD, CS and MR provided assistance and advice in conducting the statistical analyses and interpretation of the data. KR and BH, contributed to concept behind the paper, and LDM conceived the study. All authors provided input in the design of the lager study, as well as read and approved the final manuscript.

## Pre-publication history

The pre-publication history for this paper can be accessed here:

http://www.biomedcentral.com/1471-2458/13/1110/prepub
